# Miner Fatigue Detection from Electroencephalogram-Based Relative Power Spectral Topography Using Convolutional Neural Network

**DOI:** 10.3390/s23229055

**Published:** 2023-11-09

**Authors:** Lili Xu, Jizu Li, Ding Feng

**Affiliations:** 1 College of Economics and Management, Taiyuan University of Technology, Taiyuan 030024, China; xulili0124@link.tyut.edu.cn; 2 College of Coal Engineering, Shanxi Datong University, Datong 037009, China; 3 College of Data Science, Taiyuan University of Technology, Taiyuan 030024, China; fengding0173@link.tyut.edu.cn

**Keywords:** fatigue detection, convolutional neural network (CNN), the relative power spectral density (RPSD), deep learning, EEG, miners

## Abstract

Fatigue of miners is caused by intensive workloads, long working hours, and shift-work schedules. It is one of the major factors increasing the risk of safety problems and work mistakes. Examining the detection of miner fatigue is important because it can potentially prevent work accidents and improve working efficiency in underground coal mines. Many previous studies have introduced feature-based machine-learning methods to estimate miner fatigue. This work proposes a method that uses electroencephalogram (EEG) signals to generate topographic maps containing frequency and spatial information. It utilizes a convolutional neural network (CNN) to classify the normal state, critical state, and fatigue state of miners. The topographic maps are generated from the EEG signals and contrasted using power spectral density (PSD) and relative power spectral density (RPSD). These two feature extraction methods were applied to feature recognition and four representative deep-learning methods. The results showthat RPSD achieves better performance than PSD in classification accuracy with all deep-learning methods. The CNN achieved superior results to the other deep-learning methods, with an accuracy of 94.5%, precision of 97.0%, sensitivity of 94.8%, and F1 score of 96.3%. Our results also show that the RPSD–CNN method outperforms the current state of the art. Thus, this method might be a useful and effective miner fatigue detection tool for coal companies in the near future.

## 1. Introduction

Coal mine safety has attracted considerable attention in many coal-producing countries due to a significant number of accidents and a high death toll. In recent years, coal mine accidents have frequently occurred around the world, despite technological advancements and strong safety regulations. Studies have suggested that most accidents are caused by human errors of miners. Yu and Li reported that more than 85% of accidents were caused by human errors [1]. Chen, Qi, and Feng found that human errors accounted for over 95% of accidents in China [2]. Thus, reducing and mitigating human errors is an effective way to avoid similar accidents in coal mine safety management. The occurrence of human errors is generally linked to human fatigue and adverse safety accidents. Fatigue is one of the most important factors contributing to human errors. Some researchers found that human errors contribute to over 90% of mine accidents in Australia, and around 60–70% of human error accidents are related to miner fatigue [3]. The phenomenon of miner fatigue is characterized by the experience of tiredness and exhaustion among miners, which may result from prolonged and demanding work schedules, extended work hours, and irregular shift patterns. This type of fatigue can have serious effects on cognitive functions, leading to drowsiness, lack of attention, memory lapses, slow reaction times, reduced work efficiency, underestimation of risks, increased error rates, and other negative outcomes that manifest in human brain activity [4]. Therefore, the improvement of safety management in coal mines should take into account the impact of miner fatigue.

To date, many studies have focused on transport operators such as aviation and car drivers. In the past, methods for estimating driver fatigue primarily revolved around analyzing facial expression, eye tracking, and even driver behavior patterns using video and voice signals [5]. Recently, the detection of physiological signals has gained popularity as a method for estimating driver fatigue, as physiological signals offer superior performance in extracting drivers’ features and estimating fatigue detection [6]. There are different methods available to obtain information on physiological signals for detecting driver fatigue, including the electrocardiogram (ECG), electrooculography (EOG), electromyography (EMG), electroencephalogram (EEG), respiratory measurement (RM), photoplethysmography (PPG), and electrodermal activity (EDA) [7]. Among the various methods, the EEG might be one of the most popular methods for evaluating fatigue through physiological signals. Importantly, the EEG not only represents physiological signals but also captures physical activity, making it an effective method for estimating the state of fatigue.

Recently, machine-learning methods have been widely utilized in various research areas. For example, some researchers have employed the hybrid neuro-fuzzy connectionist paradigm to reliably estimate dew point pressure in retrograde gas condensate reservoirs [8]. Others have developed a machine-learning approach for the diagnosis of breast cancer using ultrasound images and contourlet transformation [9]. These studies demonstrate the effectiveness of machine-learning techniques for predictive modeling tasks. Similarly, machine-learning methods, such as the support vector machine (SVM), artificial neural network (ANN), and K-nearest neighbors (KNN) have been commonly employed by researchers for fatigue detection using EEG signals [10,11,12]. However, more recently, deep-learning methods have gained prominence over traditional machine-learning methods in fatigue detection from EEG signals. The representative deep-learning techniques [13,14,15,16] recurrent neural networks (RNNs), deep belief networks (DBNs), long short-term memory (LSTM), and convolutional neural networks (CNNs) are typically utilized to extract and combine diverse classification features from EEG signals [17,18]. In this paper, we introduce a CNN for analyzing miner fatigue, as it has shown significant success as a classifier with high accuracy. Therefore, this paper proposes the use of a CNN for miner fatigue detection using EEG signals, addressing the limitations of previous studies. Our main contributions are summarized below:

- This work contributes to our understanding of fatigue detection in mining accidents, specifically by employing deep-learning methods to assess miner fatigue. Previous research primarily focused on laboratory simulation studies or subjective questionnaire surveys concerning miner fatigue. However, there exists a knowledge gap that motivated the expansion of this research, which utilizes deep-learning techniques to measure miner fatigue;
- The RPSD–CNN method has been utilized for feature extraction and classification, leading to improved classification performance.
- CNNs have rarely been used for EEG-based fatigue classification in coal mine areas. In this respect, the third significant contribution of this work is the attempt to generate EEG signals using the proposed method;
- The fourth contribution of this work involves the detection of miner fatigue through EEG signals, which encompass both physiological and physical signals. It is the first introduced EEG model to estimate fatigue detection in coal miners. Importantly, this approach has the potential to enhance the detection ability of miner fatigue, as EEG signals contain a wealth of information that reflects the state, activities, and diseases of the brain.

The rest of the paper is organized as follows: In Section 2, we provide a literature review on fatigue detection using EEG and CNN methods. Section 3 describes the data collection, representation, and the proposed methodology. Section 4 presents the results of this research work and compares its findings with the findings of the recent works. Finally, conclusions and limitations are presented.

## 2. Related Work

Fatigue refers to a state of decreased alertness and a low level of cognitive performance. At present, the management of miner fatigue primarily involves supervising miners’ behaviors through video surveillance. However, this approach has some disadvantages. For example, it is difficult to quickly respond to emergencies, recognize complicated miners’ behaviors in a complex working environment [19], and obtain information about physiological signals. Many researchers have suggested using EEG signals to detect fatigue based on employees’ brain activity. EEG signals display the brain’s electrical activity, which is generated by the flow of ionic currents. It has been proven that EEG signals are effective for studying the human brain due to research on the spectral characteristics of electrodes [20]. It enables the monitoring of the brain activities of the driver to identify fatigue states. Fatigue can cause visible changes in EEG signals. From the above, EEG signals are introduced to automatically identify miner fatigue due to their accessibility to information in the brain [21].

### 2.1. EEG Features

In studies related to fatigue, EEG signals are generally divided into four bands of brain waves: delta, theta, alpha, and beta. Their frequency bands are as follows: delta: 0–4 Hz, theta: 4–7 Hz, alpha: 8–13 Hz, and beta: 14–30 Hz. The delta band reflects sleep quality, including sleep duration and intensity. Theta bands serve as markers of sleep propensity. Alpha bands are commonly observed in a wakeful state, which is related to relaxation, happiness, and well-being [22]. Beta bands represent a state of consciousness closely related to logical thinking [23,24]. In EEG-based fatigue detection, many researchers have excluded the delta band, and they believed that the delta band has few effects on brain activities when drivers are in the fatigue state [25]. However, some researchers have pointed out that the delta bands do exhibit visible changes during fatigue states [26]. Regarding the power of the theta band, an increasing trend between theta band and fatigue has been found [27]. Similarly, with increasing fatigue, the alpha band experiences dramatic growth. Compared to the alpha band, the beta band is believed to have better performance in fatigue detection. Some researchers have reported an increase in beta power under eyes-open conditions and a decrease under eyes-closed conditions as fatigue increases. The utilization of the four bands of EEG signals for fatigue estimation is meaningful for fatigue detection.

Several feature extraction approaches have been reported for characterizing EEG signals in each band. Power spectral density (PSD), wavelet analysis, and differential entropy (DE) are popular EEG features. PSD is widely used for EEG-based classification tasks due to its superior performance compared to other features [28]. However, it should be noted that the PSD value can vary among individuals, times, and systems, introducing potential biases. More recently, the relative power spectral density (RPSD) method has been introduced in some EEG studies, which is considered more technically robust and capable of addressing the issue of bias. In this study, in addition to PSD, RPSD is also employed for EEG feature extraction using a deep-learning method for the purpose of miner fatigue detection.

### 2.2. EEG-Based Fatigue Detection Using Deep Learning

While EEG signal features associated with machine-learning methods have achieved success in obtaining domain-invariant features, these methods are still considered low-level. Thus, obtaining high-level domain-invariant feature representations for EEG signals is desirable. Deep-learning methods provide a potential solution by producing high-level domain-invariant features and achieving high-accuracy classification results for EEG signals [29]. Representative deep-learning methods such as the RNN, LSTM, DBN, and CNN have demonstrated strong feature-learning abilities and outstanding performance in object detection and classification tasks [30,31]. As a result, deep-learning methods have gradually become a research hotspot for fatigue detection using EEG signals. Different deep-learning methods have different advantages. RNNs have proven to be effective in processing data with sequential characteristics, particularly in audio-related fields like speech recognition. LSTM is suitable for capturing time information and connecting the relationship between different channels of an EEG [32]. DBNs, which represent a more traditional deep-learning method, have been employed for classifying hyperspectral remote sensing images [33]. Researchers have widely used CNNs in image processing. Table 1 summarizes various deep-learning methods for EEG-based fatigue detection. Among various deep-learning models, most studies employed CNNs for fatigue detection of EEG signals, as CNNs are suitable for extracting features from convolution kernels and minimizing the effect of noise of EEG data. CNNs have made significant progress in different kinds of tasks due to their excellent generalization ability [34], ranging from computer vision to natural language processing [35]. Some researchers have combined multiplex recurrence networks with CNNs to extract information for driver fatigue detection [36]. Other have proposed CNN denoise networks for constructing brain networks in fatigue driving recognition [37]. Studies in the literature have generated CNNs and LSTM to construct a classification framework for EEG images [38]. Experimental results show that the CNN model commonly achieves better classification accuracy than other deep-learning models for EEG-based fatigue detection. Therefore, the CNN was selected for detecting miner fatigue in EEG signals in this study.

## 3. Materials and Methods

**Figure 1:**
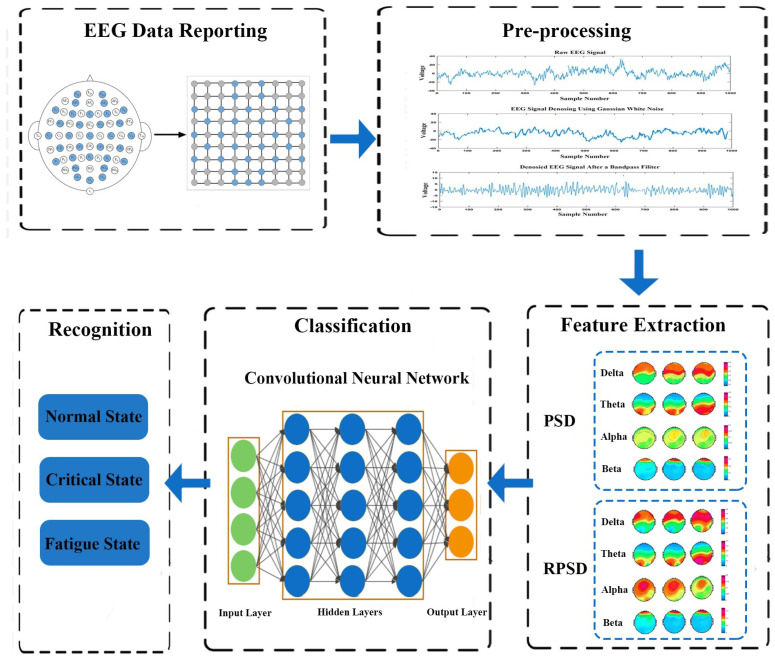
The general framework of the proposed methodology.

The general framework of the proposed methodology is shown in Figure 1. EEG signals were acquired from 32 electrodes, which were arranged according to the international 10–20 standard. Then, EEG data underwent several pre-processing procedures. Following the pre-processing procedures, the EEG signals were extracted into two-dimensional features using PSD and RPSD for each frequency band. After feature extraction, the CNN was used to classify the state of miners. Finally, the state of the miners was determined. Both the PSD–CNN and RPSD–CNN architectures comprise multiple layers of artificial neurons specifically designed for processing EEG signals. The architectures need to be configured to match the sampling rate and other characteristics of the generated EEG signals.

### 3.1. Participants and Experimental Apparatus

In this study, fifteen male miners were selected from the Tianqi Lithium Corporation, one of the most well-known mining companies in China. All the subjects agreed to participate in this study, and they ranged in age from 22 to 33. According to their self-reports, all the subjects were in good health. None of them has a sleep disorder which may cause fatigue. All participants were required to follow three guidelines: (1) Avoid drinking alcohol and taking medicines within 24 h before the experiments. (2) Avoid vigorous exercise within one hour before the experiments. (3) Avoid drinking coffee and energy drinks within 8 h before the experiments.

**Figure 2:**
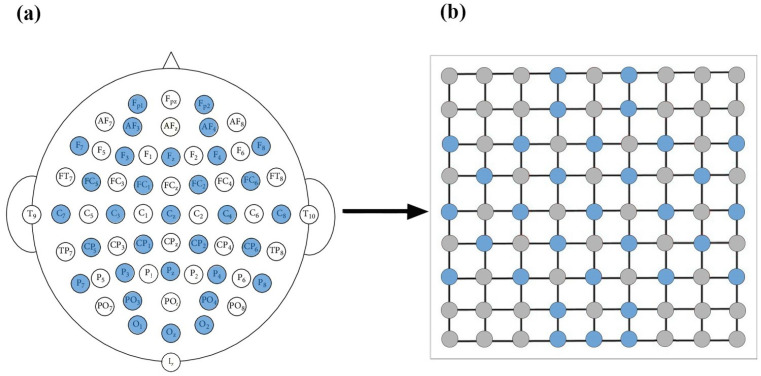
EEG input construction. (**a**) The 32-channel EEG placement in this experiment. (**b**) EEG channel matrix.

The brain–computer interface equipment used in this study comprised a hard hat that meets mining safety requirements and a wireless EEG device to collect and record raw EEG signal data. This equipment primarily collected EEG signals with 32 channels. The 32 channels are Fp1, AF3, F3, F7, FC5, FC1, C3, C7, CP5, CP1, P3, P7, PO3, O1, Oz, Pz, Fp2, AF4, Fz, F4, F8, FC6, FC2, Cz, C4, C8, CP6, CP2, P4, P8, PO4, and O2 (Figure 2). To enable the CNN model to perform optimal feature extraction, it is important that the inputs within its receptive field are spatially correlated. To achieve this, we mapped the scalp location of each channel to a specific location in a two-dimensional matrix using a data organization method based on azimuthal equidistant projection. According to this method, a matrix of 9×9 was used in this study; see the blue circled points in Figure 2. The electrodes used in the data collection process were constructed from Ag/AgCl material. For Fp1 and Fp2 sites, planar electrodes were utilized, while the remaining sites employed papillary electrodes with spiky projections. This electrode selection strategy was implemented to establish consistent and reliable contact between the electrodes and the scalp, thereby minimizing noise interference during the recording process. The hard hat uses Bluetooth to transmit data to the recording software at regular intervals of 10 min.

### 3.2. Experimental Procedure and Data Collection

Prior to the experiment, the staff provided the miners who participated in the experiment with a comprehensive explanation of the experimental purpose, procedures, and precautions. This step ensures that the miners understand the experiment. Additionally, the staff assisted the drivers in wearing the hard hat with EEG device equipment and confirmed that the records are normal. To systematically analyze the changes in the EEG signals, all the subjects were recorded from 9:00–12:00 and 13:00–18:00. During the experimental process, the staff monitored real-time data and periodically inquired about the miners’ subjective level of fatigue using the Karolinska Sleepiness Scale (KSS) at every 10 min interval. After the completion of the experiment, all the data were uploaded and stored in MATLAB 2018a.

In this study, the original dataset used consisted of raw EEG data. The raw EEG data refer to the unprocessed electrical signals recorded from the participants’ scalps using EEG electrodes. The raw EEG data were used to analyze physiological signals and identify relevant features associated with fatigue. Thirty-two channels of EEG data were recorded following the international 10–20 Montage system covering the major areas of the brain. The average resistance of bilateral papillae reference and scalp-recording electrodes was 5 k·Ohm, and the signals were recorded at a rate of 500 Hz. A 60-s time window for data truncation was utilized in this study.

### 3.3. Fatigue Evaluation Methods

The KSS, comprising a 9-point Likert scale, was used to measure the miner fatigue. In this paper, the degree of miner fatigue was divided into three states: normal state, critical state, and fatigue state, which correspond to scores of 1, 6, and 9 on the KSS, as shown in Table 2.

### 3.4. EEG Data Pre-Processing

The original EEG signals were affected by various sources of noise, such as high-frequency noise generated by electromagnetic fields from alternating current and low-frequency noise caused by blinking or muscle movements. To increase the efficiency of the proposed model, we decreased the noise through a wavelet-based denoising approach, which can be expressed as

(1) Y(t)=x(t)+sigma

where Y is added to the original EEG signal x to simulate Gaussian white noise, and sigma is the amplitude of the noise.

After the denoising step, a 1–75 bandpass filter for EEG signals was adopted to minimize drift and artifacts. Thus, EEG signals were downsampled to 200 Hz for the experiments in this work. Pre-processing EEG signals provides a significant data reduction, which consequently reduces the computational complexity. EEG signals were acquired by a portable Neuracle system which was founded in the United States and is based in the city of Sterling, Virginia.

### 3.5. Power Spectral Density

Feature extraction is necessary in the construction of a deep-learning method for a fatigue detection task. In this study, PSD and PRSD were selected to infer and analyze the functional brain network. PSD and RPSD were strongly related to fatigue and KSS scores. To estimate PSD and RPSD in EEG signal processing, a windowed function was used to analyze the characteristics of the EEG signal. Use of this windowed function leads to a lower frequency resolution but widens the dynamic range. The PSD and RPSD methods are sensitive to the choice of window length and other parameters, which could ensure the robustness of the results.

The PSD for EEG signals is commonly estimated using the autoregressive (AR) Burg method, which provides good frequency resolution and can minimize spectral losses. The AR method is based on the output sequence β(n) as a causal and discrete filter whose input is white noise. The AR method is defined as

(2) $$\beta \left(n\right)=-\sum_{k=1}^{p}a_{k}x\left(n-k\right)+u\left(n\right)$$

where $a_{k}$ are the AR coefficients, *p* is the order of the AR model, and u(n) is the white noise with variance $\sigma^{2}$.

In this work, the recursive Burg method was used to estimate AR coefficients. The method is based on reducing forward and backward prediction errors. With AF parameters calculated by the Burg algorithm, PSD estimation can be expressed as

(3) $${\hat{P}}_{BURG}\left(f\right)=\frac{{\hat{e}}_{p}}{{\left|1+\sum_{k=1}^{p}{\hat{a}}_{p}e^{-j2\pi fk}\right|}^{2}}$$

where *f* represents the frequency at which the estimated PSD is being evaluated using the Burg spectral estimator, and ${\hat{e}}_{p}$ is the total least squares error. The order p of the AR method is determined by the Akaike information criterion (AIC). In this study, the model order was taken as *p* = 10. We selected 10 orders according to cross validation. Then, the relative PSD was obtained by normalizing the PSD results for each frequency band as follows

(4) $$P_{\mathrm{relative}\;}=\frac{\sum_{f=f_{1}}^{f=f_{2}}P\left(f\right)}{\sum_{f=f_{L}}^{f=f_{H}}P\left(f\right)}$$

### 3.6. Relative Power Spectral Density

RPSD is defined as the ratio of the PSD of the frequency band to the PSD of the total frequency band. The major advantage of using relative PSD is that it could reduce the inter-individual deviation associated with absolute power due to differences between individuals in the conduction of the skull and scalp. This advantage of RPSD is that it is considered a more reliable source of information than PSD and covers the problem of PSD, which could vary among individuals, time, and system. RPSD can be expressed as

(5) $$RPSD=\frac{PSD_{\mathrm{BOI}}}{PSD_{\mathrm{total}}}$$

In order to observe the power of different rhythms, 32-channel EEG signals from all subjects were analyzed in four frequency bands.

### 3.7. Convolutional Neural Network

As we discussed before, the CNN has shown remarkable ability in computer vision tasks. The CNN is specifically designed to process pixel data and is used in image recognition and processing. The CNN has three major strengths, which are the local sensing field, weight sharing, and downsampling [46]. These three strengths can decrease the complexity of the network. The CNN model is composed of a convolutional layer, a pooling layer, and a fully connected layer. Specifically, the convolutional layer is the fundamental component of the CNN model for automatic feature extraction. The two-dimensional feature matrix is an input vector, which can be expressed as

(6) $$a=\left(\begin{array}{cccc} a_{11} & a_{12} & \ldots & a_{1n}\\ a_{21} & a_{22} & \ldots & a_{2n}\\ \ldots & \ldots & \ldots & \ldots \\ a_{m1} & a_{m2} & \ldots & a_{mn} \end{array}\right)$$

where m×n is the shape of input vector *a*. The input two-dimensional feature is convolved with filters $W_{k}$ at the convolution layer.

(7) $$W_{k}=\left(\begin{array}{c} W_{11}\\ W_{21}\\ \ldots \\ W_{i1} \end{array}\right)$$

After the convolution, output map is formed, and the feature map at the given layer is given by

(8) $$f\left(a\right)=f\left(W_{k}\times x+b_{k}\right)$$

where $W_{k}$ is the weight matrix, $b_{k}$ is the bias value, and k is the filter, which is the total number of filterings in the convolutional layer.

After each convolution layer, a rectified linear (ReLU) function is usually used as the activation function due to its reliability. It can accelerate the convergence. The ReLU function is defined by

(9) f(a)=max(0,a)

The pooling layer refers to a downsampling operation, which can reduce complexity, improve efficiency, and limit risk of over-fitting. Max pooling is used in the experiments since max pooling can be effectively used to extract features. Max pooling has the following formula

(10) $$W_{\mathrm{output}}=\frac{W_{a}-D}{S}+1$$

where $W_{\mathrm{output}}$ is max pooling output, $W_{a}$ is the function of the input volume, D is pooling window size, and S is stride.

**Figure 3:**
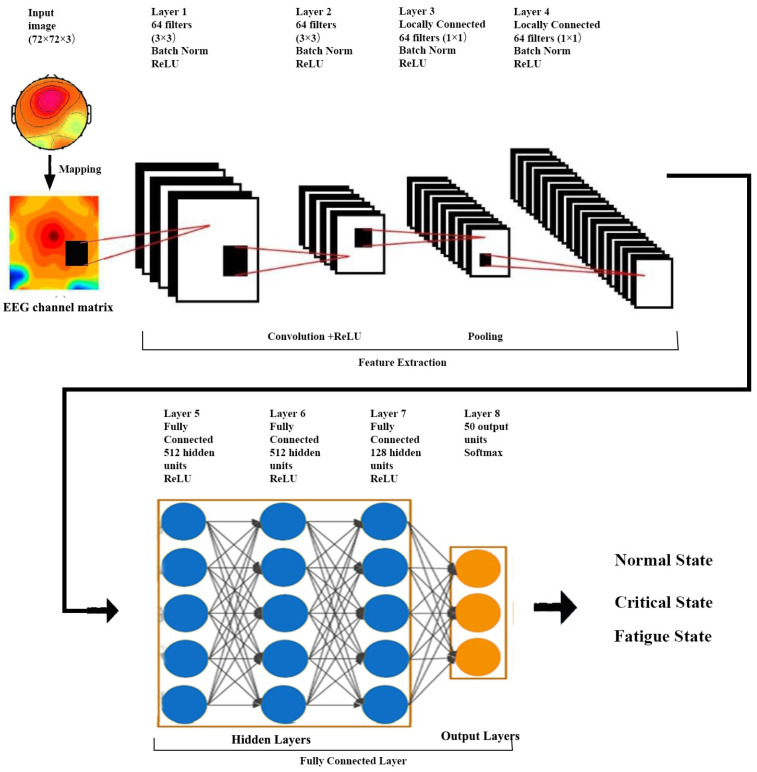
The convolutional neural network diagram.

After several convolutional and pooling layers, the output data are fully connected and typically flattened to the final outputs of the network. The fully connected layer usually maps the representation between inputs and outputs and leverages an activation function to classify inputs. In this study, the CNN composed of eight layers in total, shown in Figure 3, was based on GengNet, and the data input size was 72×72×3, where 72 is the width and 72 is the height of the image and 3 is the number of channels. We utilized zero padding to input image borders to add zero values vertically and horizontally. The output size of this designed convolutional layer can be calculated by:

(11) $$W_{\mathrm{output}}=\frac{1}{\;S+1}\left(I-\left(\left(H-1\right)\times G+1\right)+2Q\right)$$

where I is input size of image, S is stride, H is filter size, G is dilation factor, and Q is padding.

In order to optimize the CNN model, 10-fold cross validation was used for database classification. This process was repeated 10 times, with each of the subsamples used as the validation data. This could enhance the model’s generalization performance and avoid overfitting the training data. Furthermore, to improve the CNN model, the hyperparameters should be carefully selected to balance the CNN model performance with computational efficiency. In this study, 25% of the data were used for training, and the remaining 75% were used for testing. Based on the training and testing dataset, we trained and tested our proposed model using the optimum hyperparameters.

### 3.8. Performance Measures

The accuracy, precision, sensitivity, and F1 score as common estimators were selected as performance measures of different classifiers in this study. We also used these four estimators for the comparison of various classifiers. The values of the predictive instances were classified as true positive (TP), false negative (FN), true negative (TN), and false positive (FP). TP represents the number of fatigue EEG signals distinguished as fatigue states; TN indicates the number of normal EEG signals recognized as normal states; FP means the number of normal EEG signals classified as fatigue states; FN is the number of fatigue EEG signals identified as normal states. The four estimators are described and calculated as follows:

(12) $$Accuracy=\frac{TP+TN}{TP+TN+FP+FN}$$

(13) $$Precision=\frac{TP}{TP+FP}$$

(14) $$Sensitivity=\frac{TP}{TP+FN}$$

(15) $$F1=\frac{2TP}{2TP+FN+FP}$$

## 4. Experimental Results

**Figure 4:**
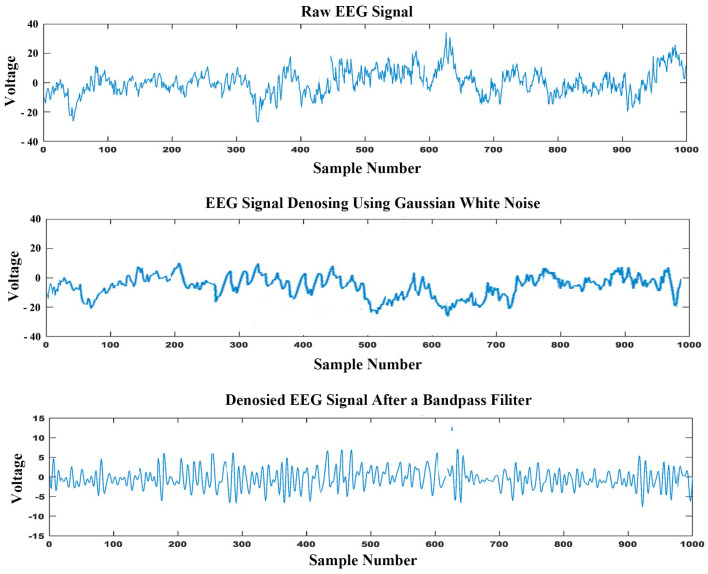
Comparison between the raw EEG signal, denoised EEG signal, and the filtered EEG signal.

All steps of the processing, data formation, feature extraction, and data classification were implemented using MATLAB 2018a. Figure 4 shows the raw EEG signal, denoised EEG, and the filtered output of the proposed reprocessing. Finally, we obtained a total of 18,300 samples. The collected data were split into training and testing sets, leaving 13,725 for training and 4575 for testing. According to the KSS score, we divided the data into normal, critical, and fatigue states. Table 3 shows the exact number of data reserved for training.

**Figure 5:**
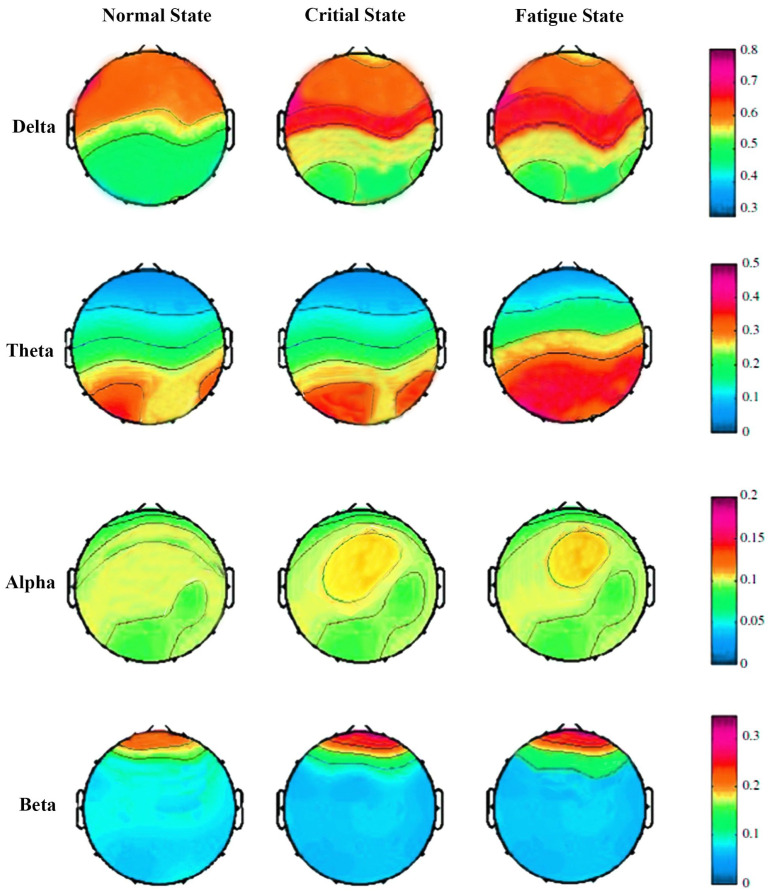
The topographic maps of PSD in the normal, critical, and fatigue states.

**Figure 6:**
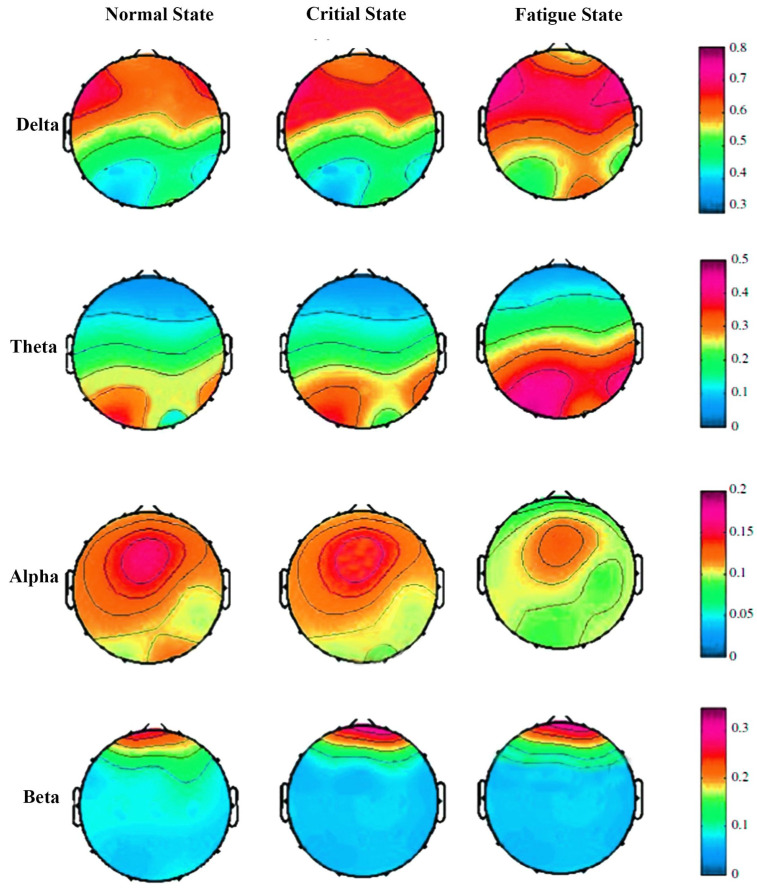
The topographic maps of RPSD in the normal, critical, and fatigue states.

The power distribution of 32-channel EEG signals using the PSD method normal state, critical state, and fatigue state in the delta, theta, alpha, and beta bands is shown in Figure 5. Visual scrutiny already reveals a significant difference among the three states, particularly in the delta and theta bands. In the theta band, the power of fatigue state is prominently enhanced compared with the normal state and critical state. Similarly, in the delta band, the PSD of EEG signals is slightly increased from the normal state to fatigue state. Comparatively, the PSD of EEG signals has no obvious change in the alpha and beta bands. The RPSD results detailed in Figure 6 suggests that a significant difference is observed in the RPSD method from the normal state to fatigue state in the delta band, theta band, and alpha band. The power of the fatigue state and critical state increased compared with the normal state for the delta band. In the theta band, the RPSD of EEG signals is gradually enhanced from the normal state to the fatigue state. However, brain activity is weakened in the alpha band. The obtained results demonstrate that the EEG power is strongly changed when miners feel fatigued during work. In the beta band, it has few changes, which indicates that fatigue may not have a strong impact on the neural processes associated with the beta band. Importantly, the power is increased in delta and theta bands but decreased in the alpha band.

The RPSD results detailed in Figure 6 suggest a significant difference in the RPSD method from the normal state to the fatigue state in the delta, theta, and alpha bands. In the delta band, both the fatigue state and critical state exhibit increased power compared to the normal state. In the theta band, the RPSD of EEG signals gradually enhances from the normal state to the fatigue state. However, brain activity is weakened in the alpha band. These findings demonstrate that the EEG power undergoes significant changes when miners experience fatigue during work. In the beta band, there are minimal changes, suggesting that fatigue may not have a strong impact on the neural processes associated with the beta band. Importantly, the power increases in the delta and theta bands but decreases in the alpha band.

**Figure 7:**
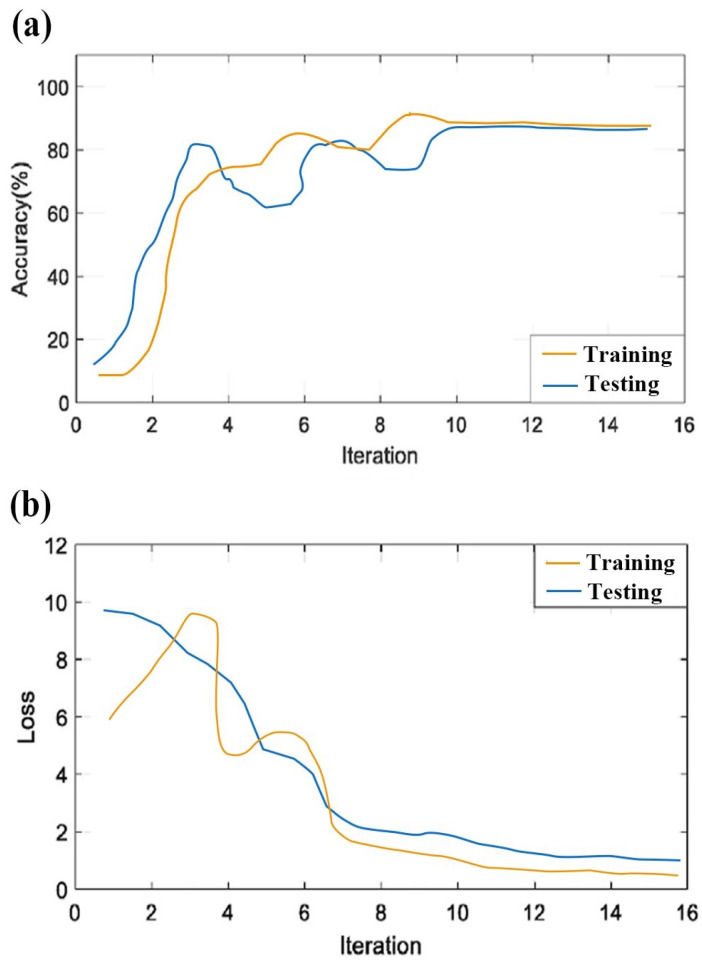
Plot of the CNN model’s accuracy and loss on training and testing. (**a**) Accuracy of the training and testing with respect to iterations; (**b**) loss of the training and testing with respect to iterations.

The topographic maps were input into the CNN for feature extraction and classification. The accuracy and loss of the proposed CNN structure for iterations are presented in Figure 7. The filter size of the convolutional layer was chosen through the trial-and-error method. Each result was examined several times, so we could select the best pattern for our experiment. All layers of the CNN were used in this work. The final results are the classification accuracies for the normal, critical, and fatigue states. The fully connected layer, thus, constructs two output classes.

The CNN model in this study is trained to detect fatigue for each subject. The individual performances were obtained on 15 subjects via 10-fold cross validation. The CNN model in this study was trained to detect fatigue for each subject. For each subject, 75% of the samples were randomly selected for training, and the remaining 25% were reserved for testing. Two feature extraction methods were used on the CNN model. Table 4 presents the performance of the CNN framework on the fatigue data using PSD and RPSD. It was found that the mean accuracy achieved was 90% for the PSD method and 94.5% for the RPSD method. This reveals that the CNN model for the RPSD method is more stable and effective on the whole dataset, and all the accuracies of the RPSD–CNN model surpass 90%.

**Figure 8:**
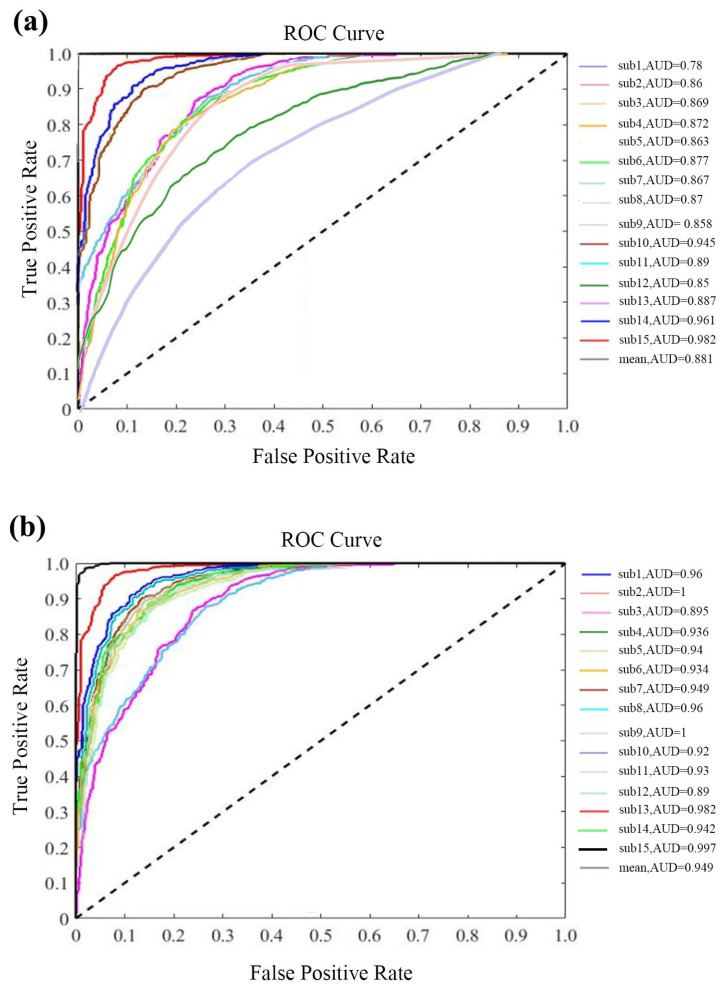
(**a**) The ROC curves and AUC values of PSD; (**b**) the ROC curves and AUC values of RPSD.

Additionally, the receiver operating characteristic (ROC) curve and area under the curve (AUC) were employed in these two scenarios to determine the validity of the proposed method. The ROC curve is the method of displaying the true positive rate (TPR) against the false positive rate (FPR) at different thresholds, which reflect sensitivity and 1-specificity values of the proposed method. Figure 8 shows the ROC curve of all subjects and the average AUC of all subjects in two scenarios. ROC analysis showed that the average AUC of the PSD–CNN method is 0.88 and is significant (*p* < 0.05). Using the RPSD method, the average AUC of the CNN is around 0.95 and is significant (*p* < 0.05). According to the results, the CNN method had a good performance for these two different feature extraction methods. Obviously, the RPSD–CNN has a better effect on fatigue detection using the proposed method. The results of the RPSD–CNN model show that the proposed method has high sensitivity and specificity in the field of fatigue detection. Considering the previous three tests of the proposed method in two scenarios, the RPSD–CNN model could gain higher accuracy, sensitivity, and specificity than the PSD–CNN model. Thus, the RPSD–CNN model was selected to measure our proposed model due to its superior classification performance.

Overfitting is a common challenge encountered in deep-learning algorithms. It occurs when a model excessively focuses on learning the intricate details of the training data, including noise, random fluctuations, and specific patterns. This narrow focus can hinder the model’s ability to generalize well for unseen data. To address this issue and evaluate the model’s performance with unseen data, it is crucial to employ techniques that help prevent overfitting. There are several techniques available to mitigate overfitting, including K-fold cross validation, regularization, dropout, and early stopping. To compare these different techniques and determine their effectiveness in preventing overfitting in deep-learning models, we utilized performance measures that capture the performance of each method. The results, as presented in Table 5, indicate that K-fold cross validation yielded the most favorable outcomes. It achieved the highest accuracy of 95.4%, the highest precision of 96.5%, the highest sensitivity of 94.2%, and the highest F1 score of 96.5%. Consequently, we selected K-fold cross validation as the preferred technique for mitigating overfitting in our study.

**Figure 9:**
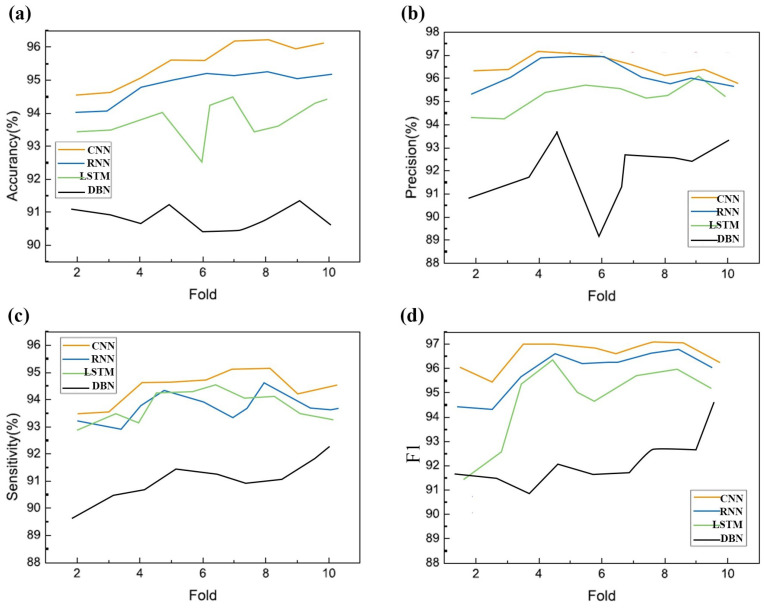
Performance plot of ten-fold cross validation vs. (**a**) accuracy; (**b**) precision; (**c**) sensitivity; (**d**) F1 for four different classifiers.

To evaluate the effectiveness of the RPSD–CNN model, we compared its performance measures with those of other representative deep-learning classifiers, namely LSTM, the RNN, and the DBN. From Table 6, it can be noted that the RPSD–CNN model achieves the highest accuracy of 96.7%, the highest precision of 97.2%, the highest sensitivity of 95.2%, and the highest F1 score of 97.2%. Similarly, the LSTM classifier demonstrates competitive performance. The accuracy ranges from 92.54% to 94.21%, precision ranges from 94.23% to 95.55%, sensitivity ranges from 92.61% to 94.31%, and F1 scores range from 91.39% to 95.23%. These results indicate that the LSTM classifier performs well in capturing temporal dependencies in the data. Moving on to the RNN classifier, accuracies ranging from 94.39% to 96.07% can be observed. The precision values range from 95.23% to 96.66%, sensitivity ranges from 92.85% to 94.24%, and F1 scores range from 94.59% to 96.63%. These results show that the RNN classifier performs consistently well across the evaluated folds. Finally, the DBN classifier exhibits slightly lower performance compared to the other classifiers. Although the DBN classifier achieves slightly lower results, it still demonstrates reasonable performance, with average accuracy, precision, sensitivity, and F1 scores all above 90%. Figure 9 summarizes the plot of classification accuracy, precision, sensitivity, and F1 of different deep-learning models for different folds in ten-fold cross validation. The CNN model outperforms other deep-learning models with 94.5%, 97%, 94.8%, and 96.3% for accuracy, precision, sensitivity, and F1, respectively. Overall, the table and figure demonstrate that the CNN classifier shows the best performance with high accuracy, precision, sensitivity, and F1 scores across the evaluated folds, indicating the effectiveness of RPSD–CNN. The LSTM classifier also performs well, although slightly worse than the CNN. The RNN and DBN classifiers, while achieving slightly lower performance, still show competitive results. Hence, it is concluded that the CNN model can have a good classification performance for EEG-based fatigue detection.

Table 7 compares the proposed work with others feature methods that use CNN classifiers. From the comparison, it can be observed that our proposed method, which achieve the highest accuracy (94.5%), outperforms most of the previous research studies. This indicates that RPSD can adapt to extract the features and form the optimal feature sets. The significance of the RPSD feature method lies in its ability to capture frequency-domain information, robustness to noise and variations, comprehensive representation of signals, and effective integration with CNN classifiers. These factors collectively contribute to achieving higher accuracy in the classification task compared to other feature methods used in previous research studies. Additionally, Table 8 compares the proposed work with other state-of-the-art methods. It shows that our proposed method outperforms most of the previous research studies in terms of accuracy. This indicates the effectiveness of the RPSD feature extraction method in combination with the CNN classifier for the given task. By capturing frequency-domain information, handling noise and variations, providing a comprehensive signal representation, and leveraging the capabilities of CNNs, the RPSD method contributes to achieving higher accuracy compared to other feature extraction methods used in previous research studies.

**Figure 10:**
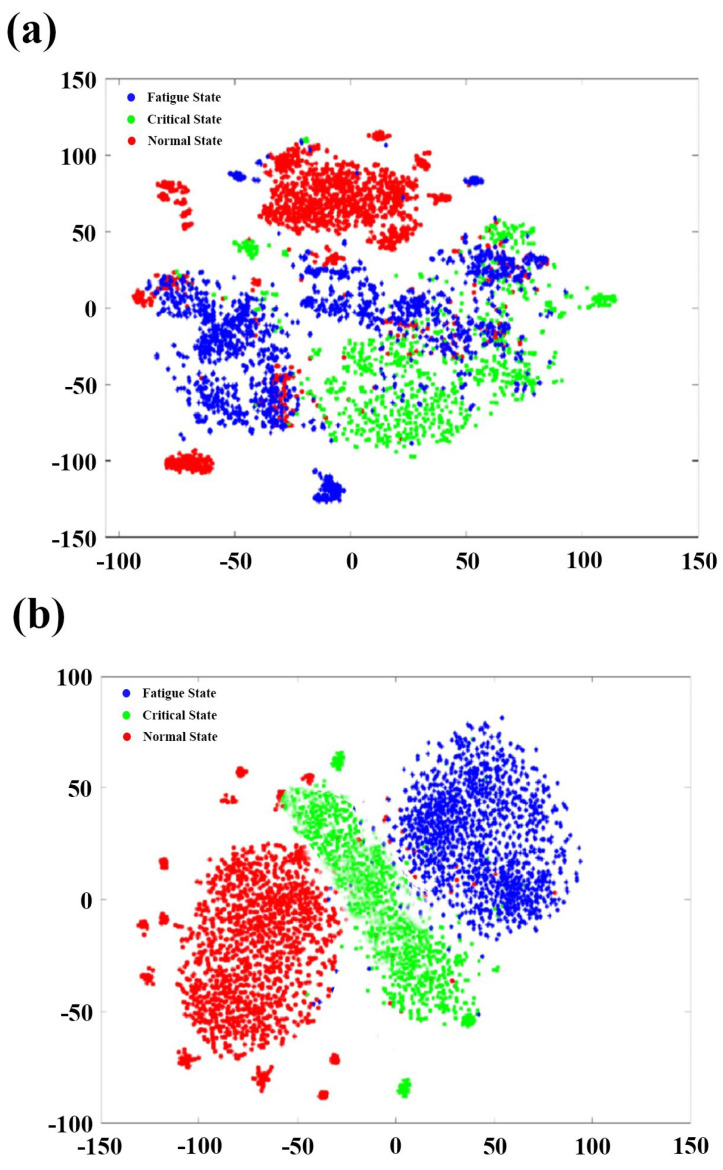
The t-SNE visualization for (**a**) the original dataset; (**b**) the data of the RPSD–CNN model.

For further analysis, t-SNE (t-Distributed Stochastic Neighbor Embedding) analysis was performed. In this study, t-SNE was used to visually compare differences before and after entering the RPSD–CNN model. As shown in Figure 10, compared to the original dataset, all the data points have clearly been gradually separated after entering the proposed model. There is no confusion between single semantic clusters after data enter the proposed model. This means that the proposed model has better feature representation, increases the separability and relative distance between single semantic clusters, and improves classification accuracy.

## 5. Discussion and Conclusions

Miner fatigue is commonly identified as an important cause of human error leading to coal mine accidents. However, the detection of miner fatigue has involved traditional methods such as supervising miners’ behaviors through video. In recent decades, EEG signals have gained attention in the analysis of driver fatigue due to the advantages of the EEG. EEG signals are more objective than vocalization, body language, or facial expression [54]. Furthermore, EEG signals are accessible. Importantly, EEG signals can effectively analyze fatigue state through physiological signals and physical activity. To estimate the miner fatigue state, multichannel EEG signals have been adopted [55]. Deep-learning models are used to promote computational models for miner fatigue detection [56]. Thus, in this study, miner fatigue has been detected from multichannel EEG signals using deep-learning models. Among different types of EEG features, we used the PSD and RPSD in EEG feature extraction, which showed better performance than other feature extraction methods in the previous studies [28]. Then, the CNN was employed as the deep-learning model to further perform feature extraction and to classify among normal, critical, and fatigue states, which can achieve a significant enhancement in the performance and can gain more representations from EEG signals. Finally, we evaluated and compared the PSD–CNN and RPSD–CNN methods, and the experimental results show that the RPSD–CNN method outperforms the other deep-learning methods. The results include 32-channel EEG signals of normal, critical, and fatigue states recorded from fifteen miners. Our results show that the EEG signals experienced substantial changes when miners feel fatigued.

Compared with the normal state, the RPSD of EEG signals in the fatigue state is highly increased in the delta and theta bands but substantially decreased in the alpha band. Some researchers also found an increase in delta and fatigue and theta and fatigue trends. But previous research reports different findings in terms of the alpha band. Some research found an increasing trend between the alpha band and fatigue [57]. However, fatigue does not lead to a noticeable decrease or increase in beta band activity. The results are different in previous studies, which have reported changes in the beta band activity with fatigue, such as a decrease in power or a shift in peak frequency. In our study, the beta band may maintain its typical level of power, frequency, and synchronization despite the presence of fatigue. This suggests that fatigue may not have a strong impact on the neural processes associated with the beta band. It is important to note that the absence of significant differences in the beta band does not negate the presence of fatigue; while the beta band did not show substantial differences, other frequency bands exhibited significant variations associated with fatigue. Further, our result reveals that the CNN achieves remarkable results due to its great success in feature representation. Our proposed method outperformed the other deep-learning methods with an average accuracy of 95.4%. The proposed methodology might become an effective method instead of traditional supervising methods for detecting miner fatigue, which could form the neurophysiology mechanisms for fatigue detection. This proposed methodology also could become a reference work for future studies or future “systems” in coal mines.

However, three major limitations of our study should be mentioned. First, this study only selected EEG signals to analyze miner fatigue. This approach is relatively limited. Future work may utilize various important physiological signals in one study. For example, EEG, EOG, and ECG signals were employed in a study to obtain information of physiological signals to detect driver fatigue. More information about the miner fatigue state may be gained with this approach. Second, the sample size is relatively small and only tested samples of 15 miners. Future research could consider examining larger samples of models for miner fatigue. Third, this study is the first to detect miner fatigue through EEG signals in China, and the lack of previous data may have caused uncertainty which impacted the experiment.

## Figures and Tables

**Table 1:** Summary of EEG-based fatigue detection papers using deep-learning methods.

Author	Year	EEG Signals	Feature Extraction	Classification Methods	Accuracy (%)
Gao et al. [34]	2019	30 channels	Spatial-temporal correlations	LSTM	91.34%
Gao et al. [36]	2019	30 channels	Recurrence network	CNN	92.95%
Sheykhivand et al. [38]	2022	32 channels	CNN	LSTM	90%
Hajinoroozi et al. [39]	2017	64 channels	Spatial correlations	CNN	86.14%
Karuppusamy et al. [40]	2020	8 channels	Wavelet transform	DNN	93.91%
Rahman et al. [41]	2021	62 channels	RPSD	CNN	94.63%
Lin et al. [42]	2021	32 channels	Noise distribution	CNN	more than 93%
Peng et al. [43]	2023	62 channels	Differential entropy	Multi-feature fusion network	85.65%
Wang et al. [44]	2023	8 channels	Continuous wavelet transform	CNN	88.85%
Gao et al. [45]	2023	21 channels	Log-Mel spectrogram	RNN	85.65%

**Table 2:** KSS subjective fatigue level description table.

KSS Level	Description	Our Study
1	Extremely alert	Normal state
2	Very alert	
3	Alert	
4	Rather alert	
5	Moderate alertness with a balanced level of wakefulness and drowsiness	
6	A little sleepy	Critical state
7	Sleepy, but can easily stay alert	
8	Sleepy, needs to work hard to stay alert	
9	Sleepy, struggles hard to stay alert	Fatigue state

**Table 3:** Data distribution for training and testing per state.

States	Training	Testing	Total
Normal state	6660	2220	8880
Critical state	4122	1374	5496
Fatigue state	2943	981	3924
Total	13,725	4575	18,300

**Table 4:** Differences in classification performance between PSD–CNN and RPSD–CNN.

Subject	PSD–CNN	RPSD–CNN
1	82.79	96.23
2	90.91	98
3	86.85	92.48
4	90.85	94.68
5	84.89	97
6	90.91	93.4
7	88.95	94.71
8	87.85	96.24
9	89.82	99
10	95.94	92.48
11	92.91	93.74
12	85.73	90
13	90.88	97.48
14	94.97	94.48
15	97.91	98.24
Average ± Standard Deviation	90.14 ± 4.82	94.51 ± 3.47

**Table 5:** Comparison with different techniques to avoid overfitting.

Techniques	Accuracy (%)	Precision (%)	Sensitivity (%)	F1 (%)
K-fold cross validation	95.41	96.48	94.21	96.48
L1 regularization	95.06	95.58	93.45	96.11
L2 regularization	95.12	95.98	93.65	96.24
Dropout	94.18	94.55	92.22	95.15
Early stopping	94.46	95.12	93.54	95.92

**Table 6:** Performance analysis of the RPSD method with the CNN, LSTM, RNN, and DBN classifier.

Folds	CNN				LSTM			
	**Accuracy (%)**	**Precision (%)**	**Sensitivity (%)**	**F1 (%)**	**Accuracy (%)**	**Precision (%)**	**Sensitivity (%)**	**F1 (%)**
1	94.39	96.23	93.21	96.39	93.19	94.43	92.61	91.39
2	94.69	96.58	93.45	96.11	93.49	94.23	92.85	91.61
3	94.73	96.38	93.65	95.45	93.55	94.08	93.45	95.15
4	95.05	97.18	94.22	97.15	93.75	94.98	92.82	95.85
5	95.45	96.98	94.54	96.92	93.85	95.32	94.14	94.62
6	95.34	96.6	94.61	96.74	92.54	95.14	94.31	94.64
7	95.81	96.31	95.03	96.61	94.21	94.71	94.23	95.21
8	96.73	96.24	95.24	96.73	93.63	94.84	93.54	95.23
9	95.78	96.44	93.85	96.58	93.88	95.55	93.05	95.18
10	96.17	95.82	94.32	96.07	94.17	94.62	92.82	94.97
**Folds**	**RNN**				**DBN**			
	**Accuracy (%)**	**Precision (%)**	**Sensitivity (%)**	**F1 (%)**	**Accuracy (%)**	**Precision (%)**	**Sensitivity (%)**	**F1 (%)**
1	94.39	95.23	93.31	94.59	91.29	90.63	89.41	91.64
2	94.51	95.41	93.15	94.61	91.11	90.8	89.65	91.51
3	94.65	95.78	92.85	94.85	90.85	91.48	90.45	91.25
4	94.75	96.58	93.62	94.85	90.55	92.18	90.62	90.85
5	95.52	96.6	94.14	96.42	91.42	92.3	91.54	92.82
6	96.04	96.66	93.61	96.14	90.54	89.1	91.21	91.64
7	95.71	96.01	93.13	96.21	90.61	92.51	90.83	91.61
8	95.83	95.74	94.24	96.63	90.73	92.34	90.94	92.43
9	95.68	95.65	93.55	96.18	91.38	92.15	91.35	92.18
10	96.07	95.52	93.72	95.97	90.57	93.02	92.02	94.37

**Table 7:** Comparing different feature extraction methods with CNN-based classification method.

Studies	Feature Methods	Classification Methods	Results
Hajinoroozi et al. [39]	Spatial correlations	CNN	86.14%
Gao et al. [36]	Recurrence network	CNN	92.95%
Lin et al. [42]	Noise distribution	CNN	93%
Zhao et al. [47]	The region of interest (ROI)	EM-CNN	93.62%
Yang et al. [48]	Multi-column	CNN	90.65%
Ed-Doughmi et al. [49]	Multi-layer model	3D-CNN	92%
Our proposed method	RPSD	CNN	94.51%

**Table 8:** Comparison between the proposed method and other previous studies.

Studies	Feature Extraction	Classification Methods	Results
Gao et al. [45]	LogMel	CRNN	85.65%
Sharma et al. [50]	HOS-LSTM	Softmax	90.81%
Wei et al. [51]	DW-CWT	SRU	80.02%
Topic and Russo [52]	CNN	SVM	88.5%
Wang et al. [53]	Differential Entropy	DNN	93.28%
Our proposed method	RPSD	CNN	94.51%

## Data Availability

Raw EEG data can be requested through a formal email.

## Abbreviations

The following abbreviations are used in this manuscript:

CNN	Convolutional neural network
EEG	Electroencephalogram
ECG	Electrocardiogram
EOG	Electrooculography
EMG	Electromyography
RM	Respiratory measurement
PPG	Photoplethysmography
EDA	Electrodermal activity
SVM	Support vector machine
ANN	Artificial neural network
KNN	K-nearest neighbors
RNN	Recurrent neural networks
DBN	Deep belief network
LSTM	Long short-term memory
ROC	Receiver operating characteristics
AUC	Area under the curve
